# Uterine Artery Embolization as an Alternative Therapeutic Option in Adenomyosis: An Observational Retrospective Single-Center Study

**DOI:** 10.3390/jcm14113788

**Published:** 2025-05-28

**Authors:** Melinda-Ildiko Mitranovici, Dan Costachescu, Dan Dumitrascu-Biris, Liviu Moraru, Laura Georgiana Caravia, Florin Bobirca, Elena Bernad, Viviana Ivan, Adrian Apostol, Ioana Cristina Rotar, Lucian Marginean

**Affiliations:** 1 Department of Obstetrics and Gynecology, Emergency County Hospital Hunedoara, 14 Victoriei Street, 331057 Hunedoara, Romania; mitranovicimelinda@yahoo.ro; 2 Department of Radiology and Medical Imaging, “Victor Babes” University of Medicine and Pharmacy, 2 Eftimie Murgu Square, 300041 Timisoara, Romania; 3 Department of Anatomy, University of Medicine, Pharmacy, Sciences and Technology “George Emil Palade”, 540142 Targu Mures, Romania; dr.dan.biris@gmail.com (D.D.-B.); lucian.marginean@umfst.ro (L.M.); 4 Faculty of Medicine, “Carol Davila” University of Medicine and Pharmacy, 050474 Bucharest, Romania; roman.laura.caravia@umfcd.ro (L.G.C.); bobirca@umfcd.ro (F.B.); 5 Department of Obstetrics and Gynecology, “Victor Babes” University of Medicine and Pharmacy, 2 Eftimie Murgu Square, 300041 Timisoara, Romania; ebernad@yahoo.com (E.B.); ivan.viviana@umft.ro (V.I.); adrian.apostol@umft.ro (A.A.); 6 Clinic of Obstetrics and Gynecology, “Pius Brinzeu” County Clinical Emergency Hospital, 300723 Timisoara, Romania; 7 Center for Laparoscopy, Laparoscopic Surgery, In Vitro Fertilization, “Victor Babes” University of Medicine and Pharmacy, 2 Eftimie Murgu Square, 300041 Timisoara, Romania; 8 Obstetrics and Gynecology I, Mother and Child Department, “Iuliu Hatieganu” University of Medicine and Pharmacy, 400012 Cluj-Napoca, Romania; cristina.rotar@umfcluj.ro

**Keywords:** adenomyosis, transvaginal ultrasound, uterine artery embolization

## Abstract

Adenomyosis is a benign gynecologic disease that mainly affects women aged 30–50 years old. **Background**: This pathology is characterized by glands and stroma of the endometrium that enter the myometrium and is confirmed through histopathological examination after hysterectomy. Transvaginal ultrasound is the most accepted imaging approach for the diagnosis and classification of adenomyosis. Existing medical treatments are not curative and are associated with several side effects. Uterine artery embolization is an alternative treatment for controlling the symptoms of adenomyosis with less trauma while preserving the uterus. **Methods**: The aim of our study was to observe the utility of uterine artery embolization (UAE) compared to hysterectomy in specific cases of adenomyosis. A retrospective cohort study was carried out between February 2024 and April 2025. We included 52 patients in our study: 27 opted for hysterectomy, while the other 25 chose to receive uterine artery embolization between January 2017 and December 2018. Clinical follow-up was assessed using a questionnaire regarding symptomatic changes in menorrhagia, pelvic pain, and quality of life before and after the surgical procedure. Statistical analyses were performed. **Results**: Patients opted for hysterectomy in cases of severe abnormal uterine bleeding before surgery that severely affected quality of life (*p* < 0.03 and *p* < 0.001). After surgery, pelvic pain improved for women who underwent UAE, but patients also reported no pelvic pain after hysterectomy. Furthermore, mild to moderate abnormal uterine bleeding was reported in cases of UAE, and bleeding stopped completely for women who had their uterus removed (*p* < 0.001). Quality of life improved for both groups and was reported as being good after the interventions. **Conclusions**: Embolization remains an alternative therapeutic option in adenomyosis but not a substitute for hysterectomy. This was concluded based on a case-by-case evaluation, depending on the desire for pregnancy, with a focus on improved clinical outcomes.

## 1. Introduction

Adenomyosis is a benign gynecologic disease [1] with a great impact on patients’ daily life and work due to its symptoms, such as chronic pelvic pain, dysmenorrhea, and menstrual disorders [2]. This disease mainly affects women aged 30–50 years old [2,3]. The definition of adenomyosis is based on the entrance of endometrial glands and stroma into the myometrium to a depth greater than 2.5 mm from the endometrial baseline [2,4]. Its pathogenesis remains unknown [4]. This process is stimulated by estrogens, which cause uterine enlargement [2]. The disease is confirmed with histopathological examination after hysterectomy. According to the Morphological Uterus Sonographic Assessment (MUSA) guidelines, ultrasound is the most accepted imaging approach for the diagnosis and classification of adenomyosis [2,5]. For now, ultrasound (USS) and magnetic resonance imaging (MRI), interpreted with MUSA criteria, are the most useful tools for the diagnosis of adenomyosis [2]. Other useful imaging tools are computed tomography (CT), hysterosalpingography (HSG), and hysteroscopy [6].

However, it remains difficult to choose the right treatment for adenomyosis [1]. There are non-surgical treatments for those who wish to preserve fertility, which include hormonal therapies such as combined oral contraceptive pills, progestins, the levonorgestrel-releasing intrauterine system, danazol, and gonadotropin-releasing hormone (GnRH) agonists and antagonists [7,8]. These treatments are not curative and are associated with several side effects such as genital atrophy, vasomotor syndrome, mood instability, and breast pain [2,8]. There is no clear boundary between adenomyotic lesions and myometrium [6,9]; thus, conservative surgical practice can lead to uterine rupture during pregnancy [2,10,11].

Uterine artery embolization (UAE) is a novel technique based on embolizing the uterine arteries and focal blood supply under the guidance of medical imaging equipment. The ectopic endometrium suffers hypoxia and ischemia, followed by necrosis. This procedure is an alternative treatment to control the symptoms [12]. This is obtained with less trauma while preserving the uterus [2].

While UAE is a well-recognized treatment for other gynecological conditions, it has also been established to be effective for symptomatic adenomyosis. There is a need to standardize the technique and clarify the utility of UAE in adenomyosis [1]. This is a new treatment that causes less trauma, followed by faster recovery and preservation of the reproductive function of the uterus; therefore, it is suitable for use in younger women to preserve fertility. UAE has a good therapeutic effect on both focal and diffuse adenomyosis, demonstrating pain relief and enhancement of quality of life [2]. The embolization technique uses gelatin sponge particles, tris-acryl gelatin microspheres (Embosphere), or polyvinyl alcohol (PVA) [13,14,15,16,17]. The non-spherical PVA particles have the disadvantage of aggregating, resulting in the obstruction of the catheter [14,16,18]. Gelatin sponge particles have the advantage of being low-cost and temporary embolic agents, reducing the need for reinterventions [14,18]. They can be used in focal or diffuse adenomyosis [14]. Serum CA125 is a good marker for evaluating patients after UAE, as it has been shown to be lower three months after the intervention [2].

The aim of our study is to observe the utility of uterine artery embolization (UAE) in specific cases of adenomyosis, chosen based on specific ultrasound characteristics and patient preference, compared to hysterectomy, which remains an optimal alternative for symptom control in this pathology.

## 2. Materials and Methods

A retrospective cohort study was carried out according to the Declaration of Helsinki between February 2025 and April 2025. The protocols for this manuscript were approved by the Ethics Committee of Al Simionescu County [3510/18.02.2025]. We used data related to the patients that were automatically recorded in the database between January 2017 and December 2018. Written informed consent to use the recorded data for studies and publications was obtained before the interventions.

We included 52 patients in our study. The inclusion criteria used in our research were prolonged or heavy menstruation, chronic pelvic pain, abdominal distention, and increased uterine volume (>56 cm). The exclusion criteria were pregnancy, allergy to iodinated contrast medium, gynecological malignancy, association with other severe diseases, or other surgical interventions. A total of 27 opted for hysterectomy, while the other 25 chose uterine artery embolization. Their decision was based on the severity of symptoms and the degree of impairment in quality of life, and some patients expressed a desire to preserve fertility.

The Quality of Life Questionnaire at baseline was completed before and after surgery. Transvaginal ultrasound (TVUS) was used for the diagnosis and to evaluate the pre- and post-surgical status. MUSA criteria were used, such as cysts in the myometrium and hyperechogenic islands, defined as adenomyotic lesions; echogenic sub-endometrial lines with irregular JZ and interrupted JZ; and trans-lesional vascularity found by Doppler ultrasound. In cases where hysterectomy was decided, adenomyosis was confirmed by histology.

For UAE, we used the modified Seldinger method, under 2% lidocaine local anesthetic. We inserted 5 French vascular catheters into the left/right femoral artery. The uterine artery can be seen as an angiographic control, and the micro-catheter was inserted into the distal end of this artery. Boston Scientific Contour, (Boston Scientific Corporation Way Marlborough, MA 01752-1234 manufactured in Alpharetta Georgia 11810 Wills Road GA3009) which contains small-sized polyvinyl alcohol particles (150 μm and 250 μm), was used for embolization. The procedure was completed when the uterine artery was completely occlusive and the catheter was removed.

The other 27 patients received hysterectomy, with antibiotic and anticoagulant prophylaxis, after 3 or 4 days of hospitalization.

Clinical follow-up was assessed using a questionnaire regarding symptomatic changes in menorrhagia, pelvic pain, and quality of life before and after the surgical procedure. The results of the 6-month follow-up were analyzed in this study. Patients received the questionnaire automatically at routine check-ups before and after the intervention. The visual analog scale (VAS) was used to describe pelvic pain. On this scale, the extremes were no pain (score of 0) and pain as bad as it could be (score of 10). The degree of pain was defined as no pain = 0, mild was 1–4, moderate was 5–7, and severe was 8–10.

The Pictorial Blood Loss Assessment Chart is a subjective assessment of the volume of blood loss during each menstrual period, based on the degree of soiling of sanitary pads. The number of pads was quantified for vaginal bleeding. Points were assigned for bleeding based on the number of pads used and were categorized as follows: zero points (no pad was used), one point (1–3 pads), two points (4–6 pads), three points (7–9 pads), four points (10–12 pads), five points (13–15 pads), six points (16–18 pads), seven points (19–21 pads), eight points (22–24 pads), nine points (25–27 pads), and ten points (≥28 pads). For quality of life assessment, we used the UFS-QOL questionnaire with 37 questions. A higher score means a better quality of life. Patients answered on a 5-point Likert scale, from “not at all”, “a little bit”, and “somewhat” to “a great deal” and “a very great deal” and from “none of the time”, “a little of the time”, and “some of the time” to “most of the time” and “all the time”. The UFS-QOL questionnaire consists of 7 subscales based on symptoms, concern, activities, control, self-consciousness, and sexual function.

After the intervention, 6 h bed rest and pressure dressing were recommended. Regular analgesia and intravenous fluids for rehydration were used post-surgery.

TVUS was also performed after treatment.

### Statistical Analysis

In the manuscript, the choice between parametric and non-parametric tests was based on the distribution of the data. Specifically, parametric tests were employed when the data followed a normal distribution, while non-parametric tests were used when the data did not meet this assumption.

The normality of the distribution of numerical variables was assessed by the Kolmogorov–Smirnov test. Numerical data are expressed as mean (standard deviation) or as median (interquartile range) for normally and non-normally distributed data, respectively.

Differences in characteristics pre- and post-interventions were compared between the two subgroups of women via the ANOVA or Kruskal–Wallis test (for numerical parametric or non-parametric data) with significance values adjusted for the number of comparisons in the post hoc tests. The Chi-squared test was used for the comparison of categorical variables. If the test indicated that the data were normally distributed (*p* > 0.05), parametric tests such as ANOVA or Kruskal–Wallis were applied. Conversely, if the data deviated from normality (*p* ≤ 0.05), non-parametric alternatives such as the Mann–Whitney U test or Kruskal–Wallis tests were used. The tables provide the overall *p*-values. Statistical analysis was performed using SPSS (IBM SPSS Statistics for Mac 2024, Version 30.0, IBM Corp: Armonk, NY, USA).

## 3. Results

In total, 25 women underwent uterine artery embolization (UAE) for adenomyosis, and 27 women opted for a hysterectomy. There was no difference in age, marital status, education, place of living, or weight. Almost half of the women in our cohort were nulliparous (Table 1).

**Figure 1:**
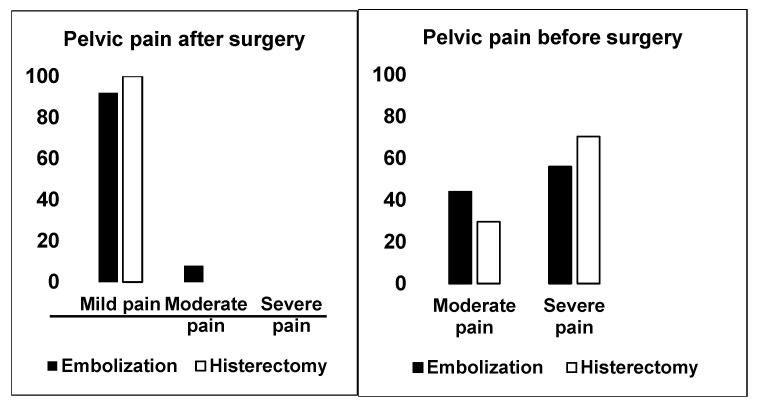
Pelvic pain before and after surgery.

The level of pelvic pain was assessed before surgery in these two groups, and more than 70% of the women who underwent hysterectomy reported a severe level of pain vs. 56% of women in the UAE group (Figure 1).

**Figure 2:**
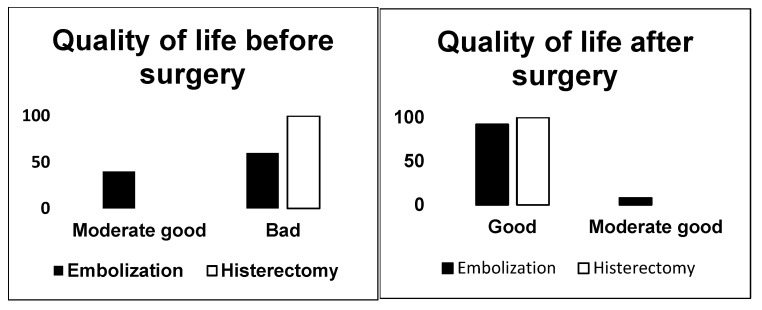
Quality of life before and after surgery.

Abnormal uterine bleeding was assessed as being moderate to severe (*p* < 0.03) in all women who had hysterectomy. Quality of life was severely affected in more than half of the patients who underwent UAE and in all the patients who opted for hysterectomy (*p* < 0.001) (Figure 2).

**Figure 3:**
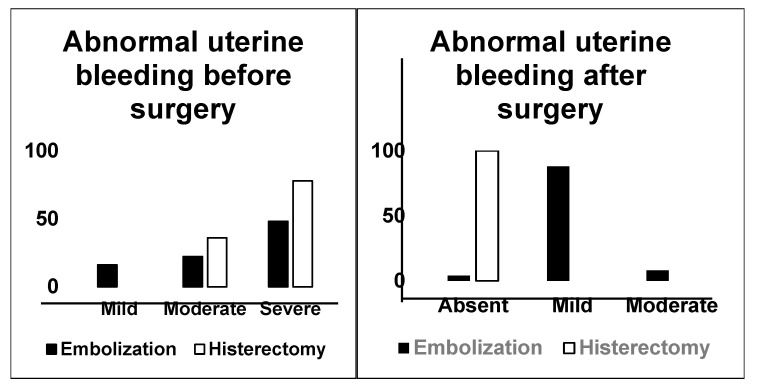
Abnormal uterine bleeding before surgery.

After surgery, pelvic pain improved for women who underwent UAE, but fewer than 1 in 10 still reported mild pain. Women who had a hysterectomy did not report any more pelvic pain. Abnormal uterine bleeding continued to be present at a mild to moderate level for more than 90 percent of women who received UAE, while it stopped completely for women who had their uterus removed (*p* < 0.001) (Figure 3).

Quality of life improved for both groups and was reported as being good after the interventions.

**Figure 4:**
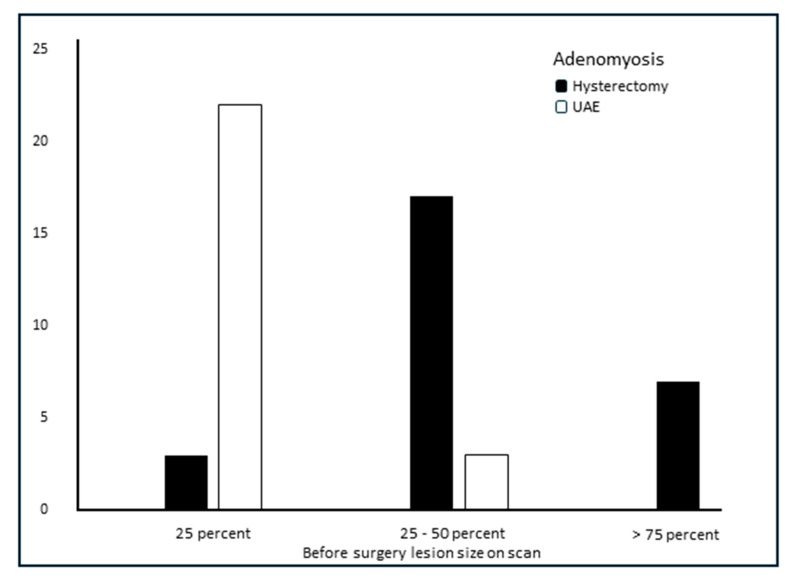
Lesion size before surgery, meaning percentage of the myometrium.

We looked at the scan characteristics before surgery in both groups of patients and noticed that the location of adenomyosis was internal in half of the cases who had UAE and in two-thirds of the cases who received a hysterectomy. A focal pattern was common for adenomyosis in UAE, and a diffuse pattern was typical for women who had a hysterectomy. An ill-defined junction zone was visible in half of the patients who had UAE and in all cases of hysterectomy (*p* < 0.001). The size of the lesion was less than 25% of the myometrium for women who had UAE in 90% of the cases and >25% for the women who had hysterectomy in 90% of the cases (*p* < 0.001) (Figure 4).

Doppler assessment before surgery showed central vascularity in all women who received UAE and none of those who had a hysterectomy. An ill-defined junction zone was seen in half of women who received UAE and in all those who had a hysterectomy (*p* < 0.001). Pre-surgery uterine artery PI was slightly higher in those who had hysterectomy (*p* < 0.02). Post-surgery, it was not possible to perform a comparison because of the nature of hysterectomy (Table 2).

Complications after surgery were systematically investigated and followed. Only one major complication was encountered after UAE, uterine necrosis, followed by hysterectomy.

## 4. Discussion

Adenomyosis poses a significant diagnostic and therapeutic challenge in women’s health because of its variety of clinical/imaging presentations and frequent coexistence with other benign gynecologic conditions. In recent years, uterine artery embolization (UAE) has shown encouraging results and favorable outcomes in the treatment of adenomyosis [19]. A multidisciplinary Research Consensus Panel was gathered by the Society of Interventional Radiology Foundation focused on imaging findings associated with symptoms, the currently available conventional treatment options, and current experiences with UAE in symptomatic adenomyosis [19].

In our study, we used imaging findings correlated with symptoms in order to identify the best surgical option. In this regard, the size of the lesion was one of the major imaging patterns that influenced the therapeutic decision: in 90% of the cases, having less than 25% of the myometrium affected was followed by UAE, whilst 50–75% of myometrium involvement led to hysterectomy (*p* < 0.001). In addition, heavy bleeding or severe pain was followed by hysterectomy. Regarding fertility preservation, even if it was desired, we have no recorded data regarding pregnancy obtained after UAE and pregnancy outcomes.

In clinical practice, hysterectomy is the most common surgical procedure used in the case of adenomyosis. Even so, it is associated with adverse mental health effects, and patients lose the opportunity to have another pregnancy [20]. However, Wei reported a good therapeutic effect in the case of UAE, where the VAS pain score, bleeding, and QOL were significantly improved [2]. In Wei’s study (2025), a total of 382 patients with adenomyosis were evaluated using the Quality of Life Questionnaire, MRI, and hemoglobin and serum CA125 after UAE with Embosphere, showing good results. Pain scores were significantly reduced, and no serious complications were encountered during the operation [2]. In our study, we used a QLQ and TVUS before and after embolization that showed an improvement in pelvic pain after surgery for both hysterectomy and UAE, but with no statistical significance. Regarding abnormal uterine bleeding, a decrease in hemorrhage was observed after UAE, while it completely stopped after hysterectomy, which made a comparison difficult. A statistically significant improvement in quality of life was observed after both interventions. One major complication after embolization was registered: necrosis, followed by hysterectomy.

Mailli in his review (2023) described an overall mean pregnancy rate in women who underwent UAE for adenomyosis of 39.4%, with a miscarriage rate of 22%, comparable to the age-matched population, meaning that this procedure can be offered to women desiring pregnancy [21]

In Yuan’s study (2021), UAE using small particles was shown to be safe and effective in conservative treatment of adenomyosis [22]. Hu described an improvement rate for dysmenorrhea of 60.4% (29/48) and decreased menorrhagia in 85.7% (30/35) of patients [23].

Pyra’s preliminary study showed patients’ overall satisfaction after UAE, describing it to be a safe and effective method [20]. Pyra reported dysmenorrhea, menorrhagia, and problems with urination in the case of adenomyosis and achieved successful embolization in all patients. The study observed a decrease in pain, a reduction in menstrual bleeding, and an improvement in everyday quality of life. Hysterectomy was avoided in 83% of patients [20].

In a study by Kim et al., one hundred and sixty-three patients underwent UAE with gelatin sponge particles for adenomyosis. They observed statistically significant differences between focal and diffuse adenomyosis, with complete necrosis with no recurrent menorrhagia or dysmenorrhea being observed in focal adenomyosis and no major complications during and after UAE [14]. In our study, necrosis was also observed in the case of diffuse adenomyosis. The TVUS performed before surgery was an essential tool for the selection of cases for UAE based on the adenomyosis pattern. UAE was considered a safe intervention for those where the size of the lesions was less than 25%, the lesions were focal, and the ill-defined junction zone was less important. Furthermore, a uterine IP of 1.2 ± 0.4 on Doppler ultrasound was another statistically significant pattern. In our study, UAE was considered a safe and useful conservative procedure. Its effectiveness in reducing bleeding was observed by Tsikouras et al. (2023) in 80–100% of cases, and pressure symptoms were reduced in 40–60% [3].

There is an important difference between fibroid embolization and adenomyosis embolization. Fibroids have peripheral and wide arterioles compared to the myometrium, and ischemia can be found in fibroids with minimal ischemic injury to the myometrium. In adenomyosis lesions, we encountered a central Doppler signal with arterioles of the same size as those found into the myometrium. This is why, with embolization, the whole myometrium becomes more ischemic, with a risk of uterine necrosis; this is the same as descriptions in the literature [3,24,25,26,27,28]. For this reason, the embolization technique in UAE for adenomyosis is conducted up to the limit of almost complete stasis, in contrast to fibroids, where complete stasis is a common technique [3]. In our study, we also used a Doppler investigation to select patients suitable for embolization. According to Liaw et al.’s (2023) study, necrotic cavitation of adenomyosis is a rare complication [29]. Morishima presented a case report about a 42-year-old woman who developed recurrent stroke with multiple cerebral infarctions after adenomyosis embolization. A systematic review identified 19 cases with cerebral infarction associated with adenomyosis where antithrombotic treatment was insufficient [30]. We did not encounter stroke in our study.

Manduca et al. (2025) obtained in his research, after embolization of adenomyosis, post-procedural improvements in menorrhagia of 89.1%, pelvic pain of 92.3%, and bulk symptoms of 97.4% [31]

The junctional zone is rarely evaluated. Turtoczki (2024), in his retrospective observational study on 577 patients, reported a significant decrease in JZ anomalies, which is a potential marker of clinical success [32]

Deipolyi et al. (2025) observed in their study that hysterectomy was the most common intervention in patients with adenomyosis, even if, compared with UAE, hysterectomy was associated with more blood transfusions, longer hospitalization, increased intestinal obstruction, and pelvic floor prolapse. But reintervention occurred in 15–20% of patients after UAE [33], while low reintervention rates were reported by Liu et al. (2023) [34].

According to Knapman’s meta-analyses conducted in 2021, there are no randomized controlled trials for UAE as a treatment for adenomyosis. Pain symptoms, menstrual bleeding, and quality of life were significantly improved, while post-procedural pain, adenomyoma, pyomyoma after necrotic cavitation of adenomyoma, Asherman syndrome, and subsequent hysterectomy were encountered as side effects [35]. As we already emphasized, we observed necrosis after one case of UAE in diffuse adenomyosis.

In Zecolla‘s review, minimally invasive interventions such as uterine artery embolization, radiofrequency ablation (RFA), percutaneous microwave ablation (PMWA) or high-intensity focused ultrasound (HIFU), and adeno-myomectomy were proven to be effective in reducing symptoms of adenomyosis. Further research is needed to assess utility and, most importantly, safety in women desiring future fertility [36].

Regarding the long-term durability of UAE for the treatment of adenomyosis, maintenance of clinical success was demonstrated in 82/91 (90%) women [37]

Studies on humans support the current pathogenic hypothesis of adenomyosis as endometrial insertion into the myometrium, although its presence at locations outside the uterus is also possible due to the development of adenomyosis from Müller’s duct [4]. Metaplastic differentiation of endometrial stem cells within the myometrium along with mutations observed in KRAS genes could also be involved in the pathogenesis of the disease [4,38,39]. Marcus described the lymphatic invasion of endometrial cells [40]. In 1972, Bird et al. defined adenomyosis as the penetration of the endometrium glands and stroma [41], which is the current definition [4,39,40,42].

The “gold” standard in adenomyosis diagnosis is histological research methods, which can be performed during hysteroscopy or laparoscopy but are mostly performed during hysterectomy [43]. Efforts have been made to diagnose adenomyosis through imaging techniques, but histopathology still remains the definitive diagnostic method [4,44]. Imaging methods have often been used for differential diagnosis [43]. Additionally, CA 125 or other markers are used, but mostly in follow-up after treatment [4].

Therefore, additional medical therapies are needed. Oral contraceptives, the levonorgestrel intrauterine device (LNG-IUS), and gonadotropin-releasing hormone agonist (GnRH-a) are options for treating adenomyosis. However, there is poor compliance with long-term oral contraceptives, side effects are important in the case of LNG-IUS, such as persistent vaginal bleeding, and menopausal symptoms have been recorded after GnRH-a. These contribute to the short-term use of medical methods. The surgical removal of adenomyosis has been encouraged, but it is difficult in the case of diffuse adenomyosis. Hysterectomy remains the most important management option for this disease. However, UAE has shown satisfactory clinical efficacy [45]. This is a relatively new procedure for organ-preserving therapy that has been used previously in fibroid treatment [3]. It is also effective in post-menopausal women with serious comorbidities [3].

UAE is a suitable alternative for patients with symptomatic adenomyosis where medical therapies have failed and in patients who wish to avoid hysterectomy. No major complications have been noted, in accordance with the Society of Interventional Radiology (SIR) classification, and good success rates have also been observed [46]. It can alleviate pain, improve fertility, and achieve therapeutic effects in patients [37,47]. UAE may cause mild uterine necrosis, which affects the endometrial functional layer, but collateral circulation can be established and the endometrium can gradually regain its normal function [2,37].

One limitation of our study is that it is observational retrospective research and there was no scope to intervene to improve the protocol. Also the lack of data regarding possible pregnancies obtained after uterine artery embolization and their evolution is a limitation of our study. The strength of this study lies in the fact that the same interventional radiologist performed the embolization in all cases and the ultrasound evaluations were performed by the same team, reducing bias and increasing the reproducibility of our research.

## 5. Conclusions

Embolization remains an alternative therapeutic option in adenomyosis, but not a substitute for hysterectomy. This conclusion was drawn based on a case-by-case evaluation, depending on desire for pregnancy and other clinical symptoms. Our aim was to emphasize the evidence supporting uterine-sparing embolization management options for adenomyotic lesions, with a focus on improved clinical outcomes. Randomized controlled trials are needed to evaluate UAE in terms of efficacy, cost, and durability of effectiveness for comparison with both medical and surgical treatments.

## Figures and Tables

**Table 1:** Maternal characteristics and comparison between various symptoms in adenomyosis before and after surgical procedures—uterine artery embolization and hysterectomy. The Pictorial Blood Loss Assessment Chart was used to measure abnormal uterine bleeding: no blood loss was 0 points, mild was 1–4, moderate was 5–7, and severe was 8–10. Quality of life using a 37-item questionnaire was defined with 148 as normal, 111–147 as good, 74–111 as mild, 37–74 as moderate, and 0–37 as severe.

Characteristics	Embolization N = 25	Hysterectomy N = 27	*p* Value
Age (y)	44 ± 5.2	45 ± 7.3	<0.45
Marital status, n (%)	23 (92)	26 (96.3)	<0.47
Education			
• elementary school	0 (0)	4 (14.8)	<0.49
• high School	18 (72)	12 (44.4)	<0.49
• university	7 (28)	11 (40.7)	<0.46
Urban, n (%)	24 (96)	23 (85.2)	<0.32
Weight (kg)	66 ± 5.8	72 ± 11.2	<0.01
Nulliparous, n (%)	10	12	<0.48
Symptoms			
Pelvic pain, n (%)			
• moderate	11 (44)	8 (29.6)	<0.21
• severe	14 (56)	19 (70.4)	<0.21
Abnormal uterine bleeding, n (%)			
• mild	4 (16)	0 (0)	<0.04
• moderate	8 (22.2)	9 (36)	<0.4
• severe	12 (48)	21 (77.8)	<0.03
Quality of life, n (%)			
• moderate good	10 (40)	0 (0)	<0.001
• bad	15 (60)	27 (100)	<0.001
Symptoms after surgery			
Pelvic pain, n (%)			
• mild	23 (92)	27 (100)	<0.22
• moderate	2 (8)	0 (0)	<0.22
• severe	0 (0)	0 (0)	-
Abnormal uterine bleeding, n (%)			
• absent	1 (4)	27 (100)	<0.001
• mild	22 (88)	0 (0)	<0.001
• moderate	2 (8)	0 (0)	<0.22
Quality of life, n (%)			
• good	23 (92)	27(100)	<0.22
• moderate good	2 (8)	0 (0)	<0.22
• no change	0 (0)	0 (0)	-

**Table 2:** Ultrasound characteristics in adenomyosis before and after surgical procedures, embolization, and hysterectomy.

Characteristics	Embolization N = 25	Hysterectomy N = 27	*p* Value
Scan before surgery			
Location, n (%)			
• internal	13 (52)	21 (77.8)	<0.48
• external	12 (48)	6 (22.2)	<0.4
Patern, n (%)			
• focal, adenomyosis	23 (92)	0 (0)	<0.001
• diffuse, adenomyosis	2 (8)	0 (0)	<0.22
• focal	0 (0)	2 (7.4)	<0.26
• diffuse	0 (0)	25 (92.6)	<0.001
Junction zone, n (%)	13 (52)	27 (100)	<0.001
Size, n (%)			
• <25%	22 (88)	3 (11.1)	<0.001
• 25–50%	3 (12)	17 (63)	<0.001
• >75%	0 (0)	7 (25.9)	<0.07
Doppler before surgery			
Central vascularity, n (%)	25 (100)	0 (0)	<0.001
Ill-defined junction zone, n (%)	13 (52)	27 (100)	<0.001
Uterine PI	1.2 ± 0.4	1.2 ± 0.5	<0.02
Doppler after surgery			
Central vascularity, n (%)	25 (100)	-	-
Ill-defined junction zone, n (%)	13 (52)	-	-
Uterine PI	0 (0)	-	-

## Data Availability

Data supporting the reported results can be found at the Department of Obstetrics and Gynecology, Emergency County Hospital Hunedoara, 14 Victoriei Street, 331057 Hunedoara, Romania.

## Abbreviations

The following abbreviations are used in this manuscript:

TVUS	transvaginal ultrasound
IP	pulsatility index
MUSA	Morphological Uterus Sonographic Assessment
MRI	magnetic resonance imaging
JZ	junctional zone
AUB	abnormal uterine bleeding
BMI	body index mass
UAE	uterine artery embolization
